# Demonstration of Cases

**Published:** 1909-10-02

**Authors:** A. H. Tubby

**Affiliations:** Surgeon, Westminster and Royal National Orthopædic Hospitals


### HOSPITAL CLINICS.

### DEMONSTRATION OF CASES.

### Dupuytrens Contraction and Ankylosis of the Hip.

By A. H. TUBBY, M.S., F.R.C.S., Surgeon, Westminster and Royal National Orthopædic Hospitals.

(Given at the Medical Graduates' College and Polyclinic, Chenies Street, W.C.).

DUPUYTREN'S CONTRACTION OF THE HAND.

GENTLEMEN,—I shall refer to this deformity because I wish to describe a method of treatment which results in many cases in cure. Dupuytren's contraction is not to be confused with other forms of contraction of the fingers. The other varieties of contraction of the fingers which one sees are due either to inflammation of the tendon sheaths, or of the joints of the fingers, such as arises from arthritis deformans, from gouty arthritis, and occasionally from gonorrhœa. Dupuytren's contraction is also confused with contraction of congenital origin.

Contraction of the palmar fascia is an affection of advanced life, usually coming on after 40 years of age. It affects all classes of society, both those who work hard with their hands, and those who are not accustomed to doing so. That is to say, it is seen in such people as carpenters, gardeners, builders, on the one side, and professional men on the other; and it is not uncommon among men-servants, such as butlers. So the idea has arisen that the contraction must necessarily be associated with gout. Inquiry shows that the gouty element is not constant.

But there is another element, namely, trauma. In 50 per cent. of the cases you will be able to elicit from the patients a history of definite trauma, either repeated or occurring on one occasion. An example of it is the following: An architect was climbing a ladder when he scratched the palm of the hand, and a small piece of mortar became embedded in it, causing some suppuration. Shortly after the wound had healed induration of the palmar fascia commenced. Another man, who had led a sedentary life, took up carpentering. After using a bradawl, and pressing vigorously with the palm of his hand, the palm remained sore, and then induration slowly commenced, and spread in the usual way. A third man forced a stick into some soft earth with his palm. He felt the hand sore, and in a few days the contraction commenced. In some traumatic cases there is a distinct history of gout. As so many cases are associated with traumatic lesions and with lesions which tear the skin, it has been thought that there must be a bacterial origin in these cases. So I have, on five occasions, had the portions of excised fascia examined, but in no case has my able colleague, Dr. Hebb, of the Westminster Hospital, detected a bacterium of any sort. In one case Mr. Lockwood described the fascia as being found encrusted with crystals of urate of soda; in most cases you will find no such thing.

With regard to its incidence, it is much more common in men than in women. In a large proportion of the cases it affects the palmar fascia opposite the root of the fourth finger, and spreads thence to the fifth finger, and from the fourth to the third finger, and so towards the thumb. But in very few cases have I seen the thumb involved. However, in two remarkable cases all the fingers and both thumbs were affected. And in two other cases I have noted an affection of the palmar fascia of both hands associated with thickening of the plantar fascia of both feet. Such an occurrence is rare indeed. One case was that of an actor, who came to me with distorted hands, and gave me the history that in each hand the affection began with general numbness and heat; and in three or four days he noticed general thickening of the fascia about the fingers, and said that with the onset in the hands there was a contraction of plantar fascia. Occasionally women are affected. I remember one case of a woman who worked in a laundry. She had an attack of gout in her right hand, and it was followed by Dupuytren's contraction.

The contraction begins in the transverse crease of the palm at the root of the ring finger. It is the part where pressure comes in using a tool, for it is the deepest part of the palm. The fascia becomes indurated, and gets gradually thicker and thicker. From that point the thickening extends downwards towards the finger, and laterally towards the adjacent digits. In the extreme cases all the fingers become involved, and in the worst, the thumb itself is affected. This contracted tissue, which is superficial, begins to undergo shortening, and so the fingers are drawn down to the palm. They are flexed at the metacarpo-phalangeal joints, then at the first, then at the second interphalangeal joint, so that finally the tip of the finger touches the palm. In some cases so extreme is the contraction that I have known the nail cause a wound in the palm of the hand. It takes 1½ to 2 years for a band of Dupuytren's contraction material to bring a finger down until it touches the palm. The ring finger having come down so far, the other fingers can no longer be fully extended, and contract until the whole hand is badly distorted. Under the skin of the palm you can feel the contracted bands of fascia; and often the skin over the bands, instead of being moist, is dry and adherent, so that you can no longer roll the skin on the subjacent tissue, and it undergoes distinct fibrotic changes.

With regard to diagnosis, Dupuytren's contraction is most often mistaken for congenital contraction of fingers; but the latter begins in the fingers themselves, whereas Dupuytren's always begins in the palm. In congenital contraction there is a thickening in the mid-line of the fingers. Dupuytren's contraction extending to the fingers on the lateral aspect. In congenital contraction there is flexion at the metacarpo-phalangeal joint, and then extension instead of flexion at the first interphalangeal joint, and afterwards flexion at the last phalangeal. And again, you have the history. Congenital contraction appears early in life, is frequently observed at birth, almost invariably affects both hands, and occasionally is accompanied by hammer-toe, especially of the second toe.

With regard to treatment, we are here dealing with contracting fibrous tissue, which shows all the qualities of ordinary scar tissue. It undergoes such contraction that no ordinary force can stem the contractile power of the tissue, so it is useless to attempt to wrench the fingers into position, or by mechanical arrangements to prevent them contracting. The operation originally advocated by Adams was that of multiple subcutaneous division of the affected fascia. He passed a fine tenotomy knife between the skin and the contracted fascia, turned the knife at right angles, and divided the fascia until he felt it give. This was done in many places at one sitting, taking care not to interfere with the branches of the digital nerves, which are found on either side of the bands. In some cases the operation was satisfactory, but in the majority, recurrence took place two or three years afterwards, and a further operation was necessary. Bearing this in mind, and having had a certain number of ill results myself from the subcutaneous method, I betook myself to the open method of treatment. It is a serious thing to open up the palm of the hand unless you are sure of your aseptic surgery, and I do not advise the operation, except in the hands of those whose technique is sound.

In people who are not too advanced in years, who have no albumen or sugar in the urine, and whose arteries are not older than their years, I invariably perform the open operation. A vertical incision is made over the mass to be removed, and by lateral incisions flaps are formed. You must use great care in dissecting back the skin that you do not buttonhole it, otherwise you will have considerable difficulty afterwards. The flaps are reflected until the limit of healthy palmar fascia is reached. When you see thin white glistening fascia you know it is healthy; but when it is thickened and cloudy you are dealing with diseased tissue, and you must go beyond it. The digital nerves are found at the roots of the fingers, and then you proceed with the dissection. Next, note the position of the palmar arch, for it is important to avoid wounding it. Then remove the whole of the diseased fascia. Sometimes it comes away in a thick mass: anything between ¼ inch and ½ inch thick. As soon as you take away the fascia, the fingers can be extended.

How should the prolongations to the fingers be dealt with? You may in some instances make flaps in the fingers, dissect, and so remove the thickened fascia in its entirety. In others, where this is not feasible, I have inserted the knife into the masses of indurated tissue, turning it round several times, and in some cases the masses have been absorbed.

You must not leave a large blood clot in the palm of the hand, therefore do not employ an Esmarch's bandage because a wound to which it is applied is very apt to bleed severely after the bandage is taken off. I advise you to pick up carefully the bleeding points and twist them; do not put any silk or foreign material into your wound. Stop the general oozing by hot water and pressure.

A new departure in treatment is this! In the majority of my cases I found the open method was often successful, still there were some relapses, especially where there was a taint of syphilis. In active infection, of course, I should not do the operation at all. The employment of fibrolysin has made the operation quite successful. I started with injecting the tissues around the wound, but that was not satisfactory, and now I rub fibrolysin into the depths of the wound, and make sure that it reaches every part of it. In addition I make five or six injections of fibrolysin into the palm, of two or three drops each around the circumference of the wound. I have found that the cases now heal perfectly; patients recover with a supple palm and extensible fingers, and all traces of induration disappear. Therefore, it appears that we have now solved the problem of the successful treatment of this troublesome affection.

### Rectangular Ankylosis of the Hip.

The case I show you offers possibilities of surgery of the most modern type; and of what one may call constructive surgery. It is one of firm ankylosis of the hip joint at right angles, on the left side. The right leg moves freely. The result of the ankylosis is that the patient walks with very great difficulty, on account of the difference in length of the two extremities. She is 27 years of age, and at the age of two and a half years she had symptoms which appear to have been those of tuberculous hip joint disease; and these symptoms resulted in the formation of an abscess, which continued to discharge until 6½ years of age. Then the abscess healed, the scars of which are seen. For 20 years she has had the deformity. What can be done to remedy it? Many years ago the late William Adams devised an operation, which is known by his name, for these cases. When the head of the femur is ankylosed, he did a subcutaneous osteotomy, and cut through the neck of the femur just below the head. The shaft of the bone can then be brought from a right angle until it is in a straight line with the trunk. Frequently there is some contraction of the psoas muscle to overcome. The late Mr. F. J. Gant devised an operation known as sub-trochanteric osteotomy, and the shaft of the femur is divided below the lesser trochanter.

Each operation has some advantages. Those of Adams' operation are that it involves very little disturbance of the parts, and, if there is a good neck, it is much more mechanically correct than the other operation, the bone being divided at its narrowest point. The disadvantages are that in nearly all cases of ankylosis of the femur there is not a well-shaped head and neck, but a stunted mass of irregular-shaped bone fixed in what remains of the acetabulum; that is to say, no definite neck at all. And those who set out to do Adams' operation, particularly after tuberculous disease of the hip joint, unless they remember certain points which I shall mention, are often landed in great difficulties. So it frequently happens that condition of the parts is not favourable to Adams'

operation; that is to say, the head has gone, and with it much of the neck. The neck of a femur in which there has been old-standing disease is intensely hard and indurated, and often calcareous, so that when you saw through it, it feels like sawing through a mass of china. The advantages of Gant's operation are that you are away from the site of the disease, and do not run any risk, as in Adams', of stirring up the old disease. And as you saw below the less trochanter, the results of your operation are not interfered with by the tonic contraction of the psoas muscle. The one disadvantage of Gant's operation is that when, after dividing the femur, you bring the lower fragment of the shaft of the femur down, you leave the lower end of the upper fragment projecting forward, and this is apt to press on the contents of Scarpa's triangle, and to cause neuralgic pains and possibly interference with the arterial circulation. What Adams set out to do was this: He took a point 1 inch above the great trochanter, passed in an osteotomy-knife to the front of neck of the femur, grooved the periosteum, then inserted his saw. The point of Adams' operation is in the passing of the saw; some people pass it much too vertically, so that they are not working at the neck of the femur, but trying to saw the remains of the acetabulum off the side of the pelvic wall. So one is not surprised that, after sawing diligently, they are glad to make a large incision to see where they are. The knife and saw must pass absolutely parallel with the outer wall of the pelvis, and at an appreciable distance from it. If you point the saw too much outwards you make a very long incision through the femur, and do an oblique trochanteric osteotomy, but it is not a bad operation. I think an oblique trochanteric osteotomy in these cases combines all the advantages of Adams' and of Gant's, and gets rid of practically all the disadvantages, because you are away from the site of the disease, avoid the salient angle, and get a good broad surface of bone for subsequent union.

In connection with this case I want to speak of another advance of great magnitude. Surgeons have lately attempted to reconstitute the movements of the hip joint, and there is already a considerable literature dealing with this subject. A French surgeon has published an excellent treatise on the subject, giving a systematic description of the steps in the operations for renewing the movements in practically every joint in the body. I have had some experience of it, and so far my results are satisfactory. Formerly, after Adams' operation, movements of the bones were made with the idea of securing a false joint. But that treatment invariably ended in failure. In order to reconstitute a joint you must have between the fragments a false bursa. And it has been found that fat is the best tissue to form these false bursæ. How do you get the fat there? It may come either from the subcutaneous tissue, or it may be formed indirectly by the fatty degeneration of muscle or fascia. Most of the operations on the hip joint have been done by inserting into the gap a flap turned down from the gluteus minimus; and in some cases the results have been successful. But modifications have been proposed, one of which (Robert Jones) is to chisel off the outer part of the trochanter major, with the gluteus minimus attached; then make a gap in the neck, and, if necessary, widen it by sawing away an additional piece of bone. Then into this bony gap insert the section from the great trochanter and fix it to the proximal surface of the neck. So there is a fibrous aponeurosis, which has been transferred and screwed on to the proximal portion of the neck, thus interposing a sufficiency of soft tissue between the two fragments.

I have recently had experience in the formation of a new elbow joint, by grafting in a large portion of the anconeus muscle, and also a part of the triceps. There was absolute ankylosis, but now the antero-posterior movements are restored, and we have succeeded in obtaining supination and pronation.

With reference to the making of new joints you have to consider carefully what type of inflammation in the joint has caused the ankylosis. In most cases it is tuberculous, in some gonorrhœal, in others streptococcic or staphylococcic infection. Of all these the tuberculous are the most favourable, and the gonorrhœal the least so for this procedure. But I think we shall find, as time goes on, that we shall be able to attack practically any and every joint which has been rendered *hors de combat* by deforming ankylosis. We have already made considerable advances in treating joints ankylosed by arthritis deformans.

